# Differentiation of Isolated Small Bowel Crohn's Disease from Other Small Bowel Ulcerative Diseases: Clinical Features and Double-Balloon Enteroscopy Characteristics

**DOI:** 10.1155/2022/5374780

**Published:** 2022-05-30

**Authors:** Meng Niu, Zheng-Hao Chen, Meng Li, Xing Zhang, Chun-Xiao Chen

**Affiliations:** ^1^Department of Gastroenterology, the First Affiliated Hospital, School of Medicine, Zhejiang University, Hangzhou, Zhejiang Province 310003, China; ^2^Department of Gastroenterology, Yiwu Fuyuan No. 1 Hospital, Yiwu, Zhejiang Province 322000, China; ^3^Department of Internal Medicine, Dongtou District People's Hospital, Wenzhou, Zhejiang Province 325000, China; ^4^Department of Gastroenterology, Affiliated Hospital of Shaoxing University (Shaoxing Municipal Hospital), Shaoxing, Zhejiang Province 312000, China

## Abstract

**Background:**

The diagnosis of isolated small bowel Crohn's disease (ISBCD) has always been challenging.

**Aims:**

This study is aimed at comparing the clinical features and double-balloon enteroscopy (DBE) characteristics of ISBCD with those of other small bowel ulcerative diseases (OSBUD).

**Methods:**

Patients with coexisting colonic and/or ileal valve lesions (*n* = 45) or whose final diagnosis was not determined (*n* = 29) were excluded. One hundred thirty-nine patients with ISBCD and 62 patients with OSBUD found by DBE were retrospectively analyzed.

**Results:**

The age of ISBCD onset was lower than that of OSBUD (OR 0.957, 95% CI 0.938-0.977, *p* < 0.001). Abdominal pain was more common in ISBCD (OR 4.986, 95% CI 2.539-9.792, *p* < 0.001). Elevated fibrinogen levels (OR 1.431, 95% CI 1.022-2.003, *p* = 0.037) and lower levels of D-dimer (OR 0.999, 95% CI 0.999-1.000, *p* = 0.017) were also more supportive of the diagnosis of ISBCD. Nonsteroidal anti-inflammatory drugs (NSAIDs) used for more than two weeks decreased the probability of a diagnosis of ISBCD (OR 0.173, 95% CI 0.043-0.695, *p* = 0.013). Abdominal computed tomography revealed a higher proportion of skip lesions in ISBCD than in OSBUD (OR 9.728, 95% CI 3.676-25.742, *p* < 0.001). The ulcers of ISBCD were more distributed in the ileum (111 (79.9%) vs. 29 (46.8%), *p* < 0.001), and their main morphology differed in different intestinal segments. Longitudinal ulcers (OR 14.293, 95% CI 4.920-41.518, *p* < 0.001) and large ulcer (OR 0.128, 95% CI 0.044-0.374, *p* < 0.001) contributed to the differentiation of ISBCD from OSBUD. We constructed a diagnostic model, ISBCD index (AUROC = 0.877, 95% CI: 0.830-0.925), using multifactorial binary logistic regression to help distinguish between these two groups of diseases.

**Conclusion:**

Clinical features, laboratory tests, abdominal computed tomography, DBE characteristics, and pathology help to distinguish ISBCD from OSBUD.

## 1. Introduction

Crohn's disease (CD) is a chronic, nonspecific inflammatory disease of unknown etiology that can affect the entire gastrointestinal tract [1]. According to previous reports, 30–70% of patients with CD have small bowel involvement, while up to 30% of patients diagnosed with CD have only small bowel involvement [2, 3]. Ulcers are the most common manifestation of small bowel CD [4, 5]. Additionally, other small bowel diseases (OSBUD), such as lymphoma, adenocarcinoma, diverticulum, tuberculosis, and Behcet's disease, also present with various forms of small intestinal ulcers [6–13]. As a result, the diagnosis of isolated small bowel Crohn's disease (ISBCD) has always been a challenge [14].

Due to the unique anatomical structure of the small intestine, it is difficult to provide an effective diagnosis for ISBCD with traditional methods, such as esophagogastroduodenoscopy, colonoscopy, X-ray examination, and high-resolution ultrasound. Computed tomography (CT) and magnetic resonance imaging have advantages in revealing transmural diseases and providing evidence of stenosis and fibrosis [15, 16]. However, lesions on the mucosal surface, such as ulcers and erosions, may be missed. Small bowel capsule endoscopy allows the direct visualization of the mucosal surface, which is of great value for monitoring disease activity. Additionally, the inability to biopsy and the potential risk of retention limit its value [17]. In contrast, double-balloon enteroscopy (DBE) is notably superior to CT and capsule endoscopy in the differential diagnosis of intraluminal small bowel diseases [18, 19].

Although there have been many studies reporting differential diagnosis of ISBCD, few of them are incorporating DBE analysis. Therefore, we thought that a systematic retrospective analysis including DBE of ISBCD and OSBUD was necessary.

## 2. Methods

### 2.1. Patients and Materials

All the procedures involving human participants were conducted in accordance with the standards of the Ethics Committee of the First Affiliated Hospital of Zhejiang University, the 1964 Declaration of Helsinki, and its later amendments or comparable ethical principles. Informed consent for the individual participant was waived since no patients were at risk in the retrospective analysis.

We collected data on patients who were hospitalized at the First Affiliated Hospital of Zhejiang University from January 01, 2013, to September 30, 2020, with small bowel ulcers detected by DBE. All patients completed a demographic survey before DBE and had it recorded in their medical records. Data included general information, medical history, physical examination, laboratory tests, radiology, DBE characteristics, and histology results. Smoking was defined as 5 or more cigarettes per day for more than 3 months, regardless of whether one was still currently smoking; alcohol consumption was defined as more than 140 g per week for men and more than 70 g per week for women for more than 3 months, regardless of whether one was still currently drinking. We recorded the location, number (1, or ≥2), morphology, depth, and concomitant manifestations of he ulcers of each patient as observed by DBE, and if an ulcer had multiple characteristics, each characteristic was recorded separately. Since there is still short of a clear definition of DBE ulcers, we drew on the definition of capsule endoscopic ulcers to generate classification in the supplementary material (available here) [20].

**Figure 1:**
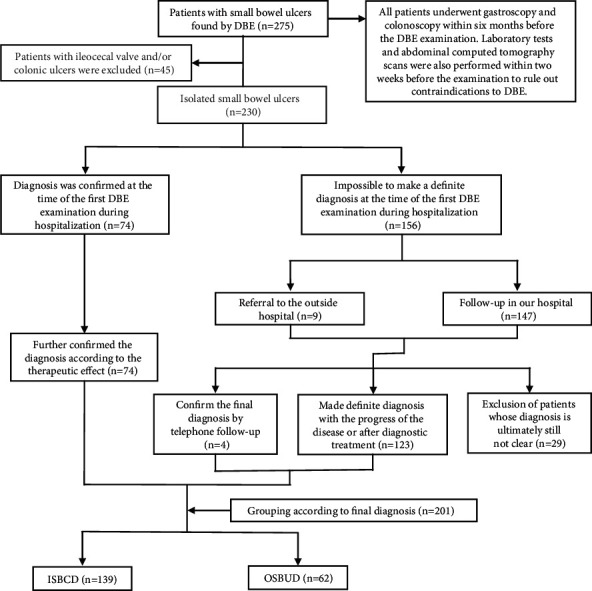
Diagnostic and grouping process. ISBCD: isolated small bowel Crohn's disease; OSBUD: other small bowel ulcerative diseases.

All patients underwent gastroscopy and colonoscopy within six months before the DBE examination. Laboratory tests and abdominal computed tomography scans were also performed within two weeks before the examination to rule out contraindications to DBE. Patients who underwent DBE review and patients who underwent DBE evaluation after small bowel transplantation were excluded. All patients (*n* = 275) with small bowel ulcers between the duodenum and ileum found by DBE were preliminarily included in the study. Then, patients with colonic and/or ileocecal valve lesions were excluded from the study (*n* = 45). Some patients were diagnosed by DBE pathological biopsy or postoperative pathology (*n* = 47); if it could not be confirmed by pathology, it would be confirmed according to the international diagnostic criteria of related diseases and the follow-up treatment effect. Therefore, all patients were followed up for at least 12 months. Patients whose final diagnosis were not determined (*n* = 29), including those with a diagnosis of nonspecific small bowel ulcer, were excluded. Finally, 139 patients with ISBCD and 62 patients with OSBUD were included in the study. The patients were divided into two groups, namely, ISBCD and OSBUD, according to their final diagnosis. The diagnosis and grouping process is detailed in Figure 1.

### 2.2. Statistics

The data were imported into SPSS 26.0 (IBM, Chicago, IL, USA) software for statistical analysis. The measurement data were first verified for normality using the Kolmogorov–Smirnov test. The data that conformed to a normal distribution are expressed as $\bar{x}\pm s$, and comparisons between groups were conducted using Student's *t*-test. The data that did not conform to a normal distribution are expressed as the median and quartiles *M* (Q1-Q3), and comparisons between groups were performed using the Mann–Whitney *U* rank-sum test. The count data are expressed as *n* and %. The *χ*^2^ test and Fisher's exact test were used for comparisons between groups, and the Bonferroni method was used for intragroup analysis. All variables were evaluated as continuous predictors in univariate analysis. The variables with a higher odds ratio (OR) were added to a multiple logistic regression model to identify independent predictors for the presence of ISBCD. To identify candidate predictors of ISBCD, we performed a stepwise logistic regression analysis (probability to enter = 0.05 and probability to remove = 0.10). A simple model using representative variables was established to predict ISBCD based on the results of multiple logistic regression analyses. The goodness of fit of the models was evaluated using the Hosmer-Lemeshow statistic. The predictive accuracy of the models for detecting ISBCD was evaluated using areas under receiver-operating characteristic curves (AUROC) with 95% confidence intervals (CI). Sensitivities and specificities of the model were also calculated. A *p* value less than 0.05 was considered statistically significant. Some data were calculated and plotted using GraphPad Prism 9 (GraphPad, San Diego, CA, USA).

## 3. Results

### 3.1. Etiological Classification

A total of 201 eligible patients with small bowel ulcers were enrolled. Of the total, ISBCD remained the main cause of small bowel ulcers in 139 patients (69.2%). There were 62 patients (30.8%) in OSBUD, including patients with lymphoma, diverticulum, cryptogenic multifocal ulcerous stenosing enteritis (CMUSE), drug-related ulcer, tuberculosis, ischemic enteropathy, eosinophilic enteritis, and polyp. The etiologies and locations of the ulcers are detailed in Table 1.

### 3.2. Demographics and Clinical Features

In ISBCD, the age of onset was 14 to 80 years, with a median of 37 (26–47) years. In OSBUD, the age of onset ranged from 20 to 80 years with a median of 48 (36–60) years. The age of onset was significantly different between the two groups (*p* < 0.001). To explore the difference, we divided the ages into the following six groups for analysis: ≤20, 21–30, 31–40, 41–50, 51–60, and ≥61 years. Between ISBCD and OSBUD, there were statistically significant differences in the ≤20, 21–30, and ≥61 year subgroups. There was no statistically significant difference in sex between the two groups (*p* = 0.852). In addition to drug-related ulcers (5 cases) in the traditional sense, 3 cases in ISBCD and 2 cases (small bowel adenocarcinoma and CMUSE) in OSBUD were also considered to be related to NSAIDs. This was because the patients developed new gastrointestinal bleeding on top of the original clinical symptoms after a period of NSAID use, and we believe that the drug use caused or promoted the development of ulcers. The proportion of patients who used nonsteroidal anti-inflammatory drugs (NSAIDs) for more than 2 weeks was higher in OSBUD (3 (2.2%) vs. 7 (11.3%), *p* = 0.016). Other general conditions were not significantly different between the two groups of patients.

Abdominal pain was the most common symptom, and it was observed significantly more frequently in ISBCD (117 (84.2%) vs. 32 (51.6%), *p* < 0.001). Diarrhea was more common in ISBCD (34 (24.5%) vs. 6 (9.7%), *p* = 0.015). A history of perianal lesions including severe hemorrhoids requiring surgical treatment, perianal abscesses, and anal fistulas was also more common in ISBCD (30 (21.6%) vs. 4 (6.5%), *p* = 0.008). Black stools (20 (14.4%) vs. 13 (35.5%), *p* = 0.001) and weakness (13 (9.4%) vs. 14 (22.6%), *p* = 0.011) were more common in OSBUD. The proportion of ISBCD patients with concomitant abdominal pain or diarrhea symptoms based on perianal lesions was significantly higher than that of OSBUD patients (22(15.8%) vs. 2 (3.2%), *p* = 0.011). Other clinical symptoms and extraintestinal manifestations were not significantly different between the two groups.  Table 2 provides demographics and clinical features.

### 3.3. Laboratory Tests

Routine blood tests showed higher platelet counts and hemoglobin and hematocrit levels in ISBCD than in OSBUD (261 (215-330) vs. 231 (191-313), *p* = 0.037; 123 (106-138) vs. 107 (84-128), *p* < 0.001; 37.9% (34.2%-41.4%) vs. 33.6% (26.6%-39.2%), *p* < 0.001, respectively). Coagulation screening showed slightly higher fibrinogen levels in patients with ISBCD than in patients with OSBUD (3.11 (2.42-3.78) vs. 2.58 (2.15-3.32), *p* = 0.005), but lower D-dimer levels than in the latter group (195 (170-459) vs. 411 (227-731), *p* < 0.001). No significant differences were found between the two groups in terms of serum total calcium, lipid metabolism, inflammation, or tumor markers.  Table 3 shows the comparisons of some of our laboratory tests.

### 3.4. Abdominal Computed Tomography

It was difficult to diagnose ulcers on the mucous surface by abdominal computed tomography. The data for only one patient in OSBUD suggested the presence of ulcer. The frequency of skip lesions in ISBCD was significantly higher than that in OSBUD (64 (46.0%) vs. 5 (8.1%), *p* < 0.001). The proportion of ISBCD patients with bowel wall thickening was higher (122 (87.8%) vs. 34 (54.8%), *p* < 0.001). There was no significant difference in the proportion of bowel strictures, intestinal wall enhancement, enhanced density of the peri-intestinal fat, mesenteric lymph node enlargement, perforation, or mesenteric abscess between the two groups, as detailed in Table 4.

### 3.5. DBE Characteristics

The choice of the DBE approach was based on the patient's clinical symptoms and other prior examination findings used to infer the location of the lesion, and if this could not be initially determined, both transoral and transanal examinations were performed. Of the 201 DBE examinations, 58 were transoral, 109 were transanal, and 34 were performed by both oral and anal approaches. The location of the ulcers was different between the two groups. Most of the ulcers in ISBCD occurred in the ileum (111 (79.9%)), while the proportion in the jejunum and ileum in OSBUD was similar (34 (54.8%) vs. 29 (46.8%)), and the probability of ulcer presence in the duodenum was lower in both groups (5 (3.6%) in ISBCD vs. 8 (12.9%) in OSBUD, *p* = 0.030). Although the proportion of multifocal ulcers was higher in the ISBCD group as a whole (94 (67.6%) vs. 29 (46.8%), *p* = 0.005), the proportion of multiple intestinal segments involved in the two groups was similar (11 (7.9%) vs. 7 (11.3%), *p* = 0.439). ISBCD differed in the terms of the morphology of major ulcers in different intestinal segments. The probability that the majority of ulcers were superficial small ulcers (3 (60.0%) vs. 18 (51.4%) vs. 20 (17.9%), *p* < 0.001) and longitudinal ulcers (0 (0%) vs. 5 (14.3%) vs. 65 (58.0%), *p* < 0.001) was different in the duodenum, jejunum, and ileum. In contrast, the main ulcer morphology in OSBUD was similar in different intestinal segments, with superficial small ulcers (5 (62.5%) vs. 17 (50.0%) vs. 15 (51.7%), *p* = 0.813), longitudinal ulcers (0 (0%) vs. 3 (8.8%) vs. 1 (3.4%), *p* = 0.766), and large ulcers (1 (21.5%) vs. 9 (26.5%) vs. 5 (17.2%), *p* = 0.541). Further analysis of the 18 patients with multiple bowel segments affected showed no significant difference in the proportion of inconsistent ulcer morphologies in different bowel segments in the same patient (9 (81.8%) vs. 5 (71.4%), *p* = 1.000).

Through the analysis of the morphology and concomitant endoscopic features of ulcers in different intestinal segments, we found the following characteristics:

1. The main morphology of ulcers in ISBCD was different in different intestinal segments, but there was no significant difference in OSBUD
2. Longitudinal ulcers in the ileum could help to identify ISBCD, but ulcers in the jejunum did not have good differential significance. No longitudinal ulcers in the duodenum were found in this study
3. Only in the jejunum, ulcers with intestinal stenosis were more likely to be ISBCD (17 (47.2%) vs. 7 (20.6%), *p* = 0.019)
4. At the individual level, the morphology of major ulcers varied from one intestinal segment to another in the same patient
5. Ulcers accompanied by mucosal hyperplasia were not the typical manifestation of ISBCD

**Figure 2:**
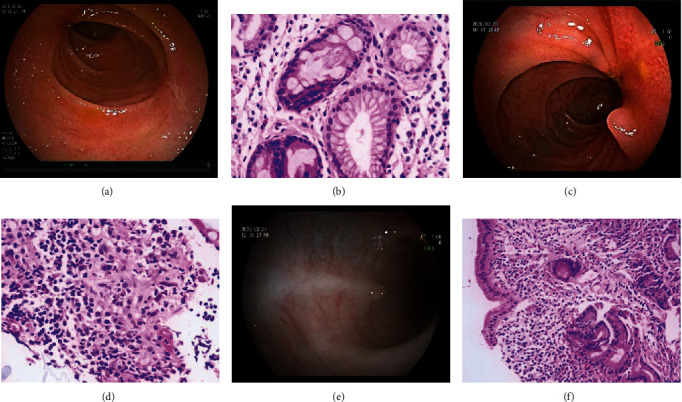
Double-balloon enteroscopy and histopathology (HE staining) of patients with isolated small bowel Crohn's disease. (a) Multiple longitudinal ulcers. (b) Pseudopyloric glandular metaplasia in the terminal ileum (×400 magnification). (c) Longitudinal ulcer with luminal stenosis. (d) Noncaseating necrotizing granuloma (×400 magnification). (e) Longitudinal ulcer scar with mucosal hyperplasia. (f) Villus atrophy of the small bowel (×200 magnification).

**Figure 3:**
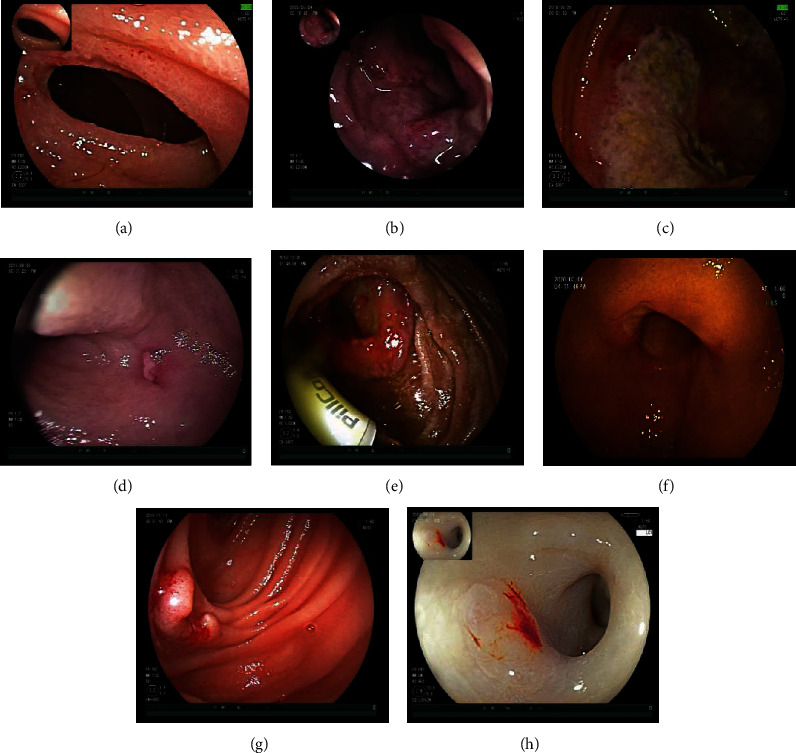
Double-balloon enteroscopy of patients with some other small bowel ulcerative diseases. (a) Cryptogenic multifocal ulcerous stenosing enteritis showed circular ulcer with luminal stenosis. (b) Behcet's disease. (c) Lymphoma. (d) Eosinophilic enteritis. (e) Adenocarcinoma with ulcers, strictures, and capsule endoscopic retention. (f) Diverticulum with ulcer. (g) Inflammatory granuloma with ulcer. (h) Drug (NSAID)-related ulcer (the mucosa was pale due to severe anemia.).

Tables 5 and 6 show the mode of entry, ulcer location, characteristics, and concomitant manifestations observed by DBE.  Figure 2 shows the manifestation and histopathology of ISBCD patients observed by DBE.  Figure 3 shows the manifestation of patients with some OSBUD as observed by DBE.

### 3.6. Pathological Histology

**Figure 4:**
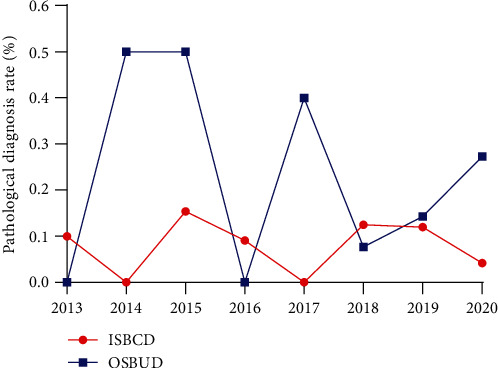
Annual change in the pathological diagnosis rate.

Pathological biopsies were collected from 127 patients (91.4%) in ISBCD and 54 patients (87.1%) in OSBUD. There was no significant difference in the proportion of biopsies collected from the two groups (*p* = 0.350). Nonnecrotic granulomas were present in 14 cases of ISBCD (13 from pathology obtained by DBE and 1 postoperative pathology) and in a case of OSBUD (patient with multiple myeloma). Although we usually consider nonnecrotic granulomas as a characteristic pathological manifestation of CD, no statistical difference was reflected between the two in our study (14 (10.1%) vs. 1 (1.6%), *p* = 0.069). Pathological histology suggested a diagnosis in a total of 11 (7.9%) patients in ISBCD and 12 (19.4%) patients in OSBUD (*p* = 0.019). Considering the improvement of pathological diagnosis technology, we further analyzed the pathological diagnosis rate in different years, and the results are shown in Figure 4. The pathology obtained by DBE biopsy has a low value for the diagnosis of ISBCD, but it has quite significant value for the differential diagnosis.

### 3.7. Derivation of the ISBCD Diagnostic Model

Univariate analysis showed that age of onset, clinical features (abdominal pain, diarrhea, weakness, black stool, history of perianal lesion, medication history of NSAIDs for more than two weeks), laboratory tests (hemoglobin, hematocrit, fibrinogen, D-dimer), abdominal computed tomography (skip lesions, bowel wall thickening), and DBE characteristics (ulcer location, superficial small ulcer, longitudinal ulcer, large ulcer, multifocal ulcer, intestinal stenosis) were significantly different between ISBCD and OSBUD. Among these variables, significant interactions were found between diarrhea, weakness, black stool, history of perianal lesion, hemoglobin, hematocrit, bowel wall thickening, ulcer location, superficial small ulcer, multifocal ulcer, and intestinal stenosis. To avoid these interactions, we incorporated representative variables with the highest ORs into the multivariate analysis. Finally, we utilized age of onset, abdominal pain, medication history of NSAIDs for more than two weeks, fibrinogen, D-dimer, skip lesions, longitudinal ulcer, and large ulcer for the multivariate analysis.

The multivariate analysis showed that age of onset (OR: 0.968, 95% CI: 0.942-0.995; *p* = 0.020), abdominal pain (OR: 3.455, 95% CI: 1.394-8.564; *p* = 0.007), medication history of NSAIDs for more than two weeks (OR: 0.087, 95% CI: 0.006-1.248; *p* = 0.072), fibrinogen (OR: 1.594, 95% CI: 0.957-2.655; *p* = 0.074), D-dimer (OR: 0.999, 95% CI: 0.999-1.000; *p* = 0.044), skip lesions (OR: 7.254, 95% CI: 2.211-23.795; *p* = 0.001), longitudinal ulcer (OR: 8.706, 95% CI: 2.360-32.120; *p* = 0.001), and large ulcer (OR: 0.194, 95% CI: 0.043-0.871; *p* = 0.032) were independent risk factors for ISBCD after adjusting for interactions between variables (Table 7). In this binary logistic regression model, the probability of having ISBCD was 1/(1 + *e*^0.358+age×0.032−abdominalpain×1.240+NSAIDsusedmorethantwoweeks×2.439−skiplesions×1.982−fibrinogen×0.466+D−dimer×0.001−longitudinalulcer×2.164+largeulcer×1.640^). We utilized the exponent of this formula and changed the multiplicative factors into the corresponding magnification coefficient. We call this formula the ISBCD index as follows: ISBCD index = abdominal pain (1, if yes) × 30 − age of onset − medication history of NSAIDs more than two weeks (1, if yes) × 10 + skip lesions (1, if yes) × 70 + fibrinogen (g/L) × 20 − D − dimer (*μ*g/L)/10 + longitudinal ulcer (1, if yes) × 80 − large ulcer (1, if yes) × 10.

**Figure 5:**
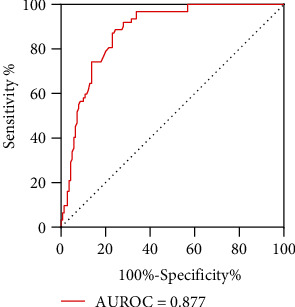
Receiver-operating characteristic (ROC) curve of ISBCD index.

The AUROC of the original formula was 0.903 (95% CI: 0.861-0.946), and the AUROC of the ISBCD index was 0.877 (95% CI: 0.830-0.925) (Figure 5). The diagnosis of ISBCD was more likely if the values were higher than the cut-off value 30.2 (Youden index = 0.642, sensitivity 75.5%, specificity 88.7%, positive likelihood ratio 6.68, negative likelihood ratio 0.28).

## 4. Discussion

Small bowel ulcers exist in many kinds of small bowel diseases. The proportion of complications increases as the disease progresses. As a result, early diagnosis is particularly important [21]. In our study, OSBUD is a collection of multiple diseases, because of the low prevalence of ulcerative disease of the small intestine and the fewer cases of related diseases, which is not sufficient for statistically significant subgroup analysis. But as the distribution of ulcers listed in Table 1, OSBUD still has similar commonalities in the distribution of ulcers. Two cases of polyps and one case of lipoma were included in OSBUD because the ulceration at the lesion resulted in not the typical polyp and lipoma presentation on DBE but rather mucosal hyperplasia-like changes, such that they were mistaken for mucosal hyperplasia due to ulceration at the initial diagnosis, but the final pathological biopsy confirmed polyps and lipoma. ISBCD (median age 37 years) was younger than OSBUD (median age 48 years). The proportion of patients with ISBCD was significantly higher among those with an age of onset of 30 years and younger. NG et al. found two peaks in the age of onset of CD, 20–24 years and 40–44 years [1]. However, in our study, we found no significant difference between the two groups in terms of the proportion of patients in the second peak interval (40–50 years). From the perspective of real-world clinical differentiation, age ≤ 30 years may be of more practical value as a diagnostic basis for ISBCD in the cluster of isolated small bowel ulcerative disease.

The symptoms of CD are heterogeneous but commonly include abdominal pain, weight loss, and chronic diarrhea [22]. A retrospective study carried out at Seoul National University Hospital observed that 41% of patients with Crohn's disease had coexisting perianal lesions [23]. In our study, there were significant differences in certain symptoms between the two groups, but it remains difficult in practice to distinguish between the two groups based on a single clinical symptom. The use of NSAIDs drugs is thought to be associated with small bowel ulcers [24]. This is further confirmed by the fact that three ISBCD and seven OSBUD patients in our study developed symptoms of gastrointestinal bleeding after more than two weeks of NSAIDs drug use.

In our study, the overall hemoglobin and hematocrit levels in ISBCD were significantly higher than those in OSBUD. Another study concluded that anemia was associated with the risk of CD [25]. Compared with our study, that study focused on the difference in data between CD patients and the general population. In patients with established small bowel ulcers, anemia may be observed more often in patients with other small bowel diseases than in patients with ISBCD. This finding is consistent with the fact that OSBUD in this study was more likely to have clinical symptoms with black stools and fatigue.

Collins et al. demonstrated that in patients with inflammatory bowel disease (IBD), platelets circulate through the mesenteric microcirculation in a highly activated state [26, 27]. During inflammatory activation, platelets induce the expression of some chemokines, complement components, and receptors for cytokines that together participate in various inflammatory responses in inflammatory bowel disease [28]. Harries et al. also noted in their study that platelet counts were significantly higher in patients with IBD than in patients with infectious diarrhea [29]. In our study, we also found higher platelet count levels in ISBCD with small bowel ulcers than in OSBUD. This finding is consistent with Li et al. [30]. Although our median platelet count level of 268 × 10^9^/L was lower than their reported level of 294.58 × 10^9^/L, this difference may be related to the selection of a specific CD population in our study.

C-reactive protein (CRP) levels have been reported to be elevated in patients with CD and to correlate with disease activity, making it a commonly used marker to assess the degree of CD activity [31, 32]. The white blood cell count, serum albumin level, and erythrocyte sedimentation rate (ESR) are commonly used indicators to reflect the nutritional status and inflammation of the body. These measures have a certain auxiliary value for the diagnosis of diseases [33, 34]. However, in our study, the differences in these indicators were not significant between the two groups of patients. This finding may be related to our choice of the diseased group as a control.

The coagulation system is an important component of the pathogenesis of IBD [35]. In our study, we found differences in fibrinogen and D-dimer between the two groups of patients. This also suggests that it is necessary to make coagulation screening a routine test for admission of CD patients. We chose these serum biomarkers for comparison and analysis because they are commonly and inexpensively detected in clinical practice.

ISBCD has DBE characteristics that distinguish it from OSBUD. In our study, both longitudinal ulcers occurring in the ileum and luminal strictures occurring in the jejunum were more likely to be found in ISBCD patients. Keuchel et al. highlighted many studies that used capsule endoscopy for the diagnosis of ulcerative disease of the small intestine and stated in their article that it is inappropriate to determine small bowel disease by the morphology of the ulcer. We remain skeptical of this argument because we believe that capsule endoscopy has limitations in the observation of ulcer morphology, especially in the holistic view (limited by the degree of intestinal lumen filling during capsule passage, interference of air bubbles and chyme, and propulsive peristalsis of the intestinal lumen), while DBE overcomes this difficulty. Therefore, it is very meaningful to identify ISBCD by summarizing the distribution, morphology, and concomitant manifestations of ulcers under DBE.

The major ulcer morphology in patients with ISBCD is different in different small bowel segments. Even at the individual patient level when multiple small bowel segments are involved, in most cases, the ulcer morphology is also different in different bowel segments. As mentioned by Coelho-Prabhu and Kane and Atreya and Siegmund in their respective articles, location played a very important role in Crohn's disease, both in terms of disease classification and in prognostic assessments [36, 37]. We believe that this characteristic is also present in ISBCD. Ulcer morphology, as an external reflection, not only is related to the type of disease but also may be related to the intestinal segment in which the ulcer is located. The reasons and mechanisms may be related to factors such as the physiological function and flora distribution of different small intestinal segments and need to be confirmed by more in-depth studies.

DBE can provide access to biopsy tissue, which can sometimes provide a critical basis for disease identification. However, we also need to realize that the pathological tissue obtained by DBE is small and superficial. ISBCD, a disease with a tendency to transmural inflammation, is often difficult to diagnose by DBE biopsies. However, this does not mean that DBE biopsy is not significant, as it is still of great interest in the differentiation of ISBCD from other diseases, such as lymphoma, intestinal tuberculosis, and adenocarcinoma [38–40]. As in our studies in 2014 and 2015, the use of DBE biopsy to obtain pathological tissue could directly diagnose 50% of OSBUD, which was difficult to do with other tests.

Considering that the diagnosis of ISBCD is difficult to determine by a specific characteristic or indicator, and to the best of our knowledge, there is no diagnostic model of ISBCD that includes DBE. We performed a multifactorial binary logistic regression analysis of the differential variables between the two groups of patients and finally derived the ISBCD index as a diagnostic model, which has positive implications for the diagnosis of ISBCD.

## 5. Conclusion

In summary, clinical features, laboratory tests, and abdominal computed tomography can all provide a basis for the diagnosis of ISBCD. DBE, as a powerful tool that allows clear and direct visualization of the mucosal surface of the small intestine, can provide very important information for the diagnosis and differential diagnosis of ISBCD. Our conclusions from this single-center retrospective study have some limitations and the possibility of bias; so, a multicenter, large sample study on the ulcerative characteristics of ISBCD observed by DBE is necessary.

## Figures and Tables

**Table 1:** Etiological classification and ulcer distribution of the 201 patients.

Etiological classification	*n* (%)	Location of ulcers (*n*)		
		Duodenum	Jejunum	Ileum
ISBCD	139 (69.2)	5	36	111
OSBUD	62 (30.8)	8	34	29
Lymphoma	16 (8.0)	0	10	7
Diverticulum	5 (2.5)	0	3	2
Drug-related ulcer	5 (2.5)	1	3	2
CMUSE	5 (2.5)	0	3	4
Stromal tumor	4 (2.0)	1	3	0
Tuberculosis	3 (1.5)	0	0	3
Eosinophilic enteritis	3 (1.5)	1	3	2
Adenocarcinoma	3 (1.5)	1	3	0
Henoch-Schonlein purpura	3 (1.5)	2	2	0
Behcet's disease	2 (1.0)	0	0	2
Ischemic bowel disease	2 (1.0)	0	0	2
Neuroendocrine carcinoma	2 (1.0)	0	2	0
Polyp	2 (1.0)	0	2	0
Inflammatory granuloma	2 (1.0)	0	0	2
Poorly differentiated carcinoma	1 (0.5)	1	0	0
Multiple myeloma	1 (0.5)	0	0	1
Parasite infection	1 (0.5)	1	0	0
Lipoma	1 (0.5)	0	0	1
Duplication of small intestine	1 (0.5)	0	0	1
Total	201 (100.0)	13	70	140

ISBCD: isolated small bowel Crohn's disease; OSBUD: other small bowel ulcerative diseases; CMUSE: cryptogenic multifocal ulcerous stenosing enteritis.

**Table 2:** Demographic and clinical features of ISBCD and OSBUD patients.

Characteristics	ISBCD	OSBUD	*p* value
	*n* (%) or median/mean	*n* (%) or median/mean	
Demographics			
Age (years, median)	37 (26–47)	48 (36–60)	<0.001
≤20	20 (14.4)^a^	1 (1.6)^b^	
21-30	35 (25.2)^a^	8 (12.9)^b^	
31-40	31 (22.3)^a^	11 (17.7)^a^	
40-50	24 (17.3)^a^	16 (25.8)^a^	
51-60	18 (12.9)^a^	11 (17.7)^a^	
≥61	11 (7.9)^a^	15 (24.2)^b^	
Sex (male, *n* (%))	96 (69.1)	42 (67.7)	0.852
Disease duration (months, median)	12.0 (2.0-36.0)	6.0 (1.8-24.0)	0.183
Medication history of NSAIDs for more than two weeks (*n* (%))	3 (2.2)	7 (11.3)	0.016
BMI (kg/m^2^, median)	20.52 (19.03-22.84)	21.30 (19.05-23.59)	0.145
Education level			0.090
Illiteracy	8 (5.8)	0 (0)	
Primary education	99 (71.2)	51 (82.3)	
Higher education	32 (23.0)	11 (17.7)	
History of tumor in immediate family	14 (10.1)	10 (16.1)	0.221
Smoking	39 (28.1)	19 (30.6)	0.708
Drinking	19 (13.7)	5 (8.1)	0.258
Blood type			0.347
A	48 (34.5)	16 (25.8)	
B	40 (28.8)	17 (27.4)	
O	37 (26.6)	24 (38.7)	
AB	14 (10.1)	5 (8.1)	
History of perianal lesion (*n* (%))	30 (21.6)	4 (6.5)	0.008
History of appendectomy (*n* (%))	16 (11.5)	3 (4.8)	0.135
General symptoms (*n* (%))			
Fever	15 (10.8)	5 (8.1)	0.551
Weakness	13 (9.4)	14 (22.6)	0.011
Weight loss	47 (33.8)	17 (27.4)	0.369
Gastrointestinal symptoms (*n* (%))			
Abdominal pain	117 (84.2)	32 (51.6)	<0.001
Diarrhea	34 (24.5%)	6 (9.7)	0.015
Black stool	20 (14.4)	13 (35.5)	0.001
Nausea and vomiting	44 (31.7)	14 (22.6)	0.190
Abdominal distention	31 (22.3)	13 (21.0)	0.833
Bloody stool	17 (12.2)	5 (8.1)	0.382
Ileus	11 (7.9)	2 (3.2)	0.348
Mucous stool	5 (3.6)	1 (1.6)	0.753
Abdominal mass	4 (2.9)	0 (0)	0.422
Abdominal discomfort	3 (2.2)	0 (0)	0.554
Extra-intestinal manifestations (*n* (%))			
Oral ulcers	44 (31.7)	13 (21.0)	0.121
Genital ulcers	6 (4.3)	4 (6.5)	0.770
Joint pain	25 (18.0)	10 (16.1)	0.749
Skin lesions	11 (7.9)	5 (8.1)	1.000
Episcleritis	6 (4.3)	1 (1.6)	0.583

^a^ and ^b^ are the subgroups that differed by Bonferroni correction.

**Table 3:** Laboratory tests.

Items	ISBCD (*n* = 139)	OSBUD (*n* = 62)	*p* value
Hemoglobin (g/L)	123 (106-138)	107 (84-128)	<0.001
Hematocrit (%)	37.9 (34.2-41.4)	33.6 (26.6-39.2)	<0.001
D-dimer (*μ*g/L)	195 (170-459)	411 (227-731)	<0.001
Fibrinogen (g/L)	3.11 (2.42-3.78)	2.58 (2.15-3.32)	0.005
Platelet count (×10^9^/L)	261 (215-330)	231 (191-313)	0.037
White blood cell count (×10^3^/L)	5.2 (4.3-6.5)	5.3 (3.8-6.6)	0.616
Neutrophil ratio (%)	64.0 ± 0.9	64.0 ± 1.5	0.985
Lymphocyte ratio (%)	25.4 ± 0.8	25.0 ± 1.3	0.752
Mean corpusular volume (fl)	86.8 (84.2-92.4)	85.8 (82.4-90.4)	0.179
Total calcium (mmol/L)	2.15 ± 0.01	2.12 ± 0.02	0.088
Uric acid (*μ*mol/L)	297 (239-359)	300 (236-351)	0.691
Albumin (g/L)	39.2 ± 0.5	38.7 ± 0.7	0.540
Total cholesterol (mmol/L)	3.56 (3.02-4.00)	3.65 (3.22-4.08)	0.526
Triglycerides (mmol/L)	0.97 (0.76-1.38)	1.04 (0.82-1.38)	0.576
CEA (ng/mL)	1.3 (0.9-1.9)	1.6 (1.0-2.3)	0.067
CRP (mg/L)	3.80 (1.26-16.0)	1.60 (0.55-19.70)	0.165
ESR (mm/60 min)	10 (6-20)	8 (3-19)	0.273
Ferritin (ng/mL)	52.2 (14.1-148.3)	60.7 (11.1-195.8)	0.731

CEA: carcinoembryonic antigen; CRP: C-reactive protein; ESR: erythrocyte sedimentation rate.

**Table 4:** Abdominal computed tomography.

Characteristics	ISBCD (*n* (%))	OSBUD (*n* (%))	*p* value
Skip lesions	64 (46.0)	5 (8.1)	<0.001
Bowel wall thickening	122 (87.8)	34 (54.8)	<0.001
Bowel strictures	44 (31.7)	19 (20.6)	0.887
Mural hyperenhancement	29 (20.9)	14 (22.6)	0.784
Ulcers	0 (0)	1 (1.6)	0.308
Enhanced density of the peri-intestinal fat	11 (7.9)	5 (8.1)	1.000
Enlarged lymph nodes	41 (29.5)	23 (37.1)	0.285
Fistula	7 (5.0)	0 (0)	0.167
Perforation	2 (1.4)	0 (0)	1.000
Abscess	2 (1.4)	0 (0)	1.000

**Table 5:** Entry mode and ulcer distribution.

	ISBCD (*n* (%))	OSBUD (%)	*p* value
Entry mode			<0.001
Transoral	22 (15.8)^a^	36 (58.1)^b^	
Transanal	92 (66.2)^a^	17 (27.4)^b^	
Both oral and anal	25 (18.0)^a^	9 (14.5)^a^	
Duodenum	5 (3.6)	8 (12.9)	0.030
Jejunum	36 (25.9)	34 (54.8)	<0.001
Ileum	111 (79.9)	29 (46.8)	<0.001
Multifocal ulcer	94 (67.6)	29 (46.8)	0.005
Multiple intestinal segments involved	11 (7.9)	7 (11.3)	0.439

^a^ and ^b^ are the subgroups that differed by Bonferroni correction.

**Table 6:** Characteristics of ulcers in different intestinal segments.

Characteristics	Duodenum (*n* (%))			Jejunum (*n* (%))			Ileum (*n* (%))		
	ISBCD	OSBUD	*p* value	ISBCD	OSBUD	*p* value	ISBCD	OSBUD	*p* value
Longitudinal ulcer	0 (0)	0 (0)	—	5 (13.9)	3 (8.8)	0.772	65 (58.6)	1 (1.5)	<0.001
Superficial small ulcer	3 (60.0)	5 (62.5)	1.000	18 (50.0)	17 (50.0)	1.000	20 (18.0)	15 (51.7)	<0.001
Large ulcer	0 (0)	1 (12.5)	1.000	0 (0)	9 (26.5)	0.003	5 (4.5)	5 (17.2)	0.049
Circular ulcer	0 (0)	0 (0)	—	6 (16.7)	5 (14.7)	0.822	17 (15.3)	8 (27.6)	0.124
Irregular ulcer	0 (0)	0 (0)	—	3 (8.3)	0 (0)	0.258	6 (5.4)	3 (10.3)	0.589
Ulcer scar	0 (0)	1 (12.5)	1.000	7 (19.4)	1 (2.9)	0.073	11 (9.9)	0 (0)	0.168
Linear ulcer	2 (40.0)	0 (0)	0.128	0 (0)	1 (2.9)	0.486	6 (5.4)	1 (3.4)	1.000
Deep ulcer	0 (0)	1 (12.5)	1.000	0 (0)	4 (11.8)	0.109	2 (1.8)	0 (0)	1.000
Intestinal stenosis	0 (0)	0 (0)	—	17 (47.2)	7 (20.6)	0.019	50 (45.0)	11 (37.9)	0.491
Mucosal hyperplasia	0 (0)	0 (0)	—	5 (13.9)	2 (5.9)	0.473	28 (25.2)	4 (13.8)	0.192

**Table 7:** Univariate and multivariate binary logistic regression for ISBCD.

Variable	Univariate model			Multivariate model		
	*χ* ^2^	OR (95% CI)	*p* value	*χ* ^2^	OR(95% CI)	*p* value
Age of onset	17.944	0.957 (0.938-0.977)	<0.001	5.452	0.968 (0.942-0.995)	0.020
Abdominal pain	21.766	4.986 (2.539-9.792)	<0.001	7.166	3.455 (1.394-8.564)	0.007
Medication history of NSAIDs for more than two weeks	6.122	0.173 (0.043-0.695)	0.013	3.228	0.087 (0.006-1.248)	0.072
Skip lesions	20.996	9.728 (3.676-25.742)	<0.001	10.687	7.254 (2.211-23.795)	0.001
Fibrinogen (g/L)	4.348	1.431 (1.022-2.003)	0.037	3.203	1.594 (0.957-2.655)	0.074
D-dimer (*μ*g/L)	5.736	0.999 (0.999-1.000)	0.017	4.051	0.999 (0.999-1.000)	0.044
Longitudinal ulcer	23.898	14.293 (4.920-41.518)	<0.001	10.556	8.706 (2.360-32.120)	0.001
Large ulcer	14.107	0.128 (0.044-0.374)	<0.001	4.583	0.194 (0.043-0.871)	0.032

## Data Availability

All the data can be found in this manuscript or received from the corresponding author upon reasonable request.

## Abbreviations

ISBCD: Isolated small bowel Crohn's disease
DBE: Double-balloon enteroscopy
OSBUD: Other small bowel ulcerative diseases
CD: Crohn's disease
CMUSE: Cryptogenic multifocal ulcerous stenosing enteritis
CT: Computed tomography
IBD: Inflammatory bowel disease
CEA: Carcinoembryonic antigen
CRP: C-reactive protein
ESR: Erythrocyte sedimentation rate
CI: Confidence interval.

## Supplementary Materials

Supplementary Materials we drew on the definition of capsule endoscopic ulcers to generate classification in the supplementary material.
