# Supplementary material for: Differentiation of Isolated Small Bowel Crohn's Disease from Other Small Bowel Ulcerative Diseases: Clinical Features and Double-Balloon Enteroscopy Characteristics
Source: Gastroenterol Res Pract. 2022 May 30;2022:5374780. doi: 10.1155/2022/5374780 (PMC9170512; doi:10.1155/2022/5374780)
Supplement: Supplementary Materials — we drew on the definition of capsule endoscopic ulcers to generate classification in the supplementary material. [file 5374780.f1.docx]

**Supplementary material**

**Differentiation of Isolated Small Bowel Crohn’s Disease from Other Small Bowel Ulcerative Diseases: Clinical Features and Double-Balloon Enteroscopy Characteristics**

Meng Niu^1,2^, Zheng-Hao Chen^3^, Meng Li^1^, Xing Zhang^4^, Chun-Xiao Chen^1^

**^1^** Department of Gastroenterology, The First Affiliated Hospital, School of Medicine, Zhejiang University, Hangzhou 310003, Zhejiang Province, China.

^2^Department of Gastroenterology, Yiwu Fuyuan No.1 Hospital, Yiwu 322000, Zhejiang Province, China.

^3^Department of Internal Medicine, Dongtou District People’s Hospital, Wenzhou 325000, Zhejiang Province, China.

^4^Department of Gastroenterology, Affiliated Hospital of Shaoxing University (Shaoxing Municipal Hospital), Shaoxing 312000, Zhejiang Province, China.

**Supplementary table**

Table 1. Ulcer classification and characteristics of DBE

| Nomenclature | Description |
| --- | --- |
| Mucosal erosion | Diminutive loss of epithelial layering with a whitish center and a red halo surrounded by normal mucosa |
| Deep ulcer | Deep loss of tissue compared to the surrounding swollen/edematous mucosa, with a whitish base, and a depth exceedings one closed biopsy forceps |
| Superficial small ulcer | Mild depressed tissue loss with a white base and features inconsistent with previously defined mucosal erosions or deep ulcers, along with a rounded or round-like appearance with a length less than that of a fully opened biopsy forceps |
| Large ulcer | The ulcer fits the description above and is round or round-like, with a long diameter larger than the opening of fully open biopsy forceps |
| Longitudinal ulcers | The long diameter of the ulcer follows the lumen path, is significantly larger than the transverse diameter (and the transverse diameter is more than half of the closed biopsy clamp), and extends longitudinally through at least two small bowel folds |
| Circular ulcer | The long axis of the ulcer is perpendicular to the direction of the intestinal lumen, annular or semiannular with a transverse diameter within two small intestinal folds |
| Irregular ulcer | Morphology cannot be described by common shapes |
| Ulcer scar | Scar-like changes with no observable small intestinal villi on the surface |
| Linear ulcer | A linear or fissure-like appearance with a very short transverse diameter (no more than half of the closed biopsy forceps) and a long-axis direction that does not necessarily follow the lumen path, usually no more than two small bowel folds |
| Intestinal stenosis | Narrowing of the intestinal lumen that did not return to normal size even after adequate gas injection and repeated pushing and pulling of the scope body |
| Mucosal hyperplasia | Abnormal mucosal hyperplasia that appeared pebble-like, polyp-like, or similar to other irregular shapes but different from a mass |
| Multiple intestinal segments involved | Ulcers involving at least two of the duodenum, jejunum, and ileum |
| Multifocal ulcers | The number of ulcers was ≥2, regardless of whether multiple intestinal segments were involved |
